# The Biomarkers in Extreme Longevity: Insights Gained from Metabolomics and Proteomics

**DOI:** 10.7150/ijms.98778

**Published:** 2024-10-21

**Authors:** Xiaorou Qiu, Yixian Lu, Chao Mu, Peihua Tang, Yueli Liu, Yongmei Huang, Hui Luo, Jun-Yan Liu, Xuemeng Li

**Affiliations:** 1 Zhanjiang Key Laboratory of Human Microecology and Clinical Translation Research, the Marine Biomedical Research Institute, College of Basic Medicine, Guangdong Medical University, Zhanjiang, Guangdong, 524023, China.; 2 Guangdong Key Laboratory of Age-Related Cardiac and Cerebral Diseases, Affiliated Hospital of Guangdong Medical University, Zhanjiang, 524000, China.; 3 CNTTI of the Institute of Life Sciences & Anesthesia Department of the Second Affiliated Hospital, Chongqing Medical University, Chongqing, 400016, China.; 4 Basic Medicine Research and Innovation Center for Novel Target and Therapeutic Intervention, Chongqing Medical University, Chongqing, 400016, China.

**Keywords:** Biomarkers, Longevity, Metabolomics, Proteomics, Multi-omics

## Abstract

The pursuit of extreme longevity is a popular topic. Advanced technologies such as metabolomics and proteomics have played a crucial role in unraveling complex molecular interactions and identifying novel longevity-related biomarkers in long-lived individuals. This review summarizes key longevity-related biomarkers identified through metabolomics, including high levels of omega-3 polyunsaturated fatty acids (PUFAs), short-chain fatty acids (SCFAs) and sphingolipids, as well as low levels of tryptophan. Proteomics analyses have highlighted longevity-related proteins such as apolipoprotein E (APOE) and pleiotrophin (PTN), along with lower S-nitrosylated and higher glycosylated proteins found from post-translational modification proteomics as potential biomarkers. We discuss the molecular mechanisms that could support the above biomarkers' potential for healthy longevity, including metabolic regulation, immune homeostasis maintenance, and resistance to cellular oxidative stress. Moreover, multi-omics studies of various long-lived cohorts are encompassed, focusing on how the integration of various omics technologies has contributed to the understanding of longevity. This comprehensive review aims to provide new biological insights and pave the way for promoting health span.

## 1. Introduction

The ongoing trend of global aging indicates that human life expectancy will continue to rise 1. The average life expectancy in 2019 was 72.6 years old and is expected to rise to 77.1 years old in 2050 according to the World Population Prospects (United Nations, 2019). However, the elderly may be affected by age-related diseases, so the central problem of aging is to explain why the organism cannot adapt to these endogenous stimuli, such as oxidative stress and molecular hyperfunction 2. Instead, we believe that a central question of longevity is to explain why organisms are able to delay or avoid adverse stimuli. Hence, extending the human lifespan while achieving healthy aging to longevity simultaneously are the major goals of global aging and anti-aging research. The healthy elderly over 90 years old, who are the representatives of extreme longevity, have reached the limit of human longevity while largely avoiding and postponing major age-related diseases, thereby making them the most successful examples of healthy aging 3, 4. As individuals who are deemed most likely to achieve extreme longevity, the offspring of healthy longevity have the unique interests of researchers 5, 6. Therefore, the identification of potential longevity-related biomarkers in centenarians and their offspring and the exploration of the complex associations among biomarkers are critical for extending lifespan.

During the journey to seeking longevity biomarkers, metabolites and proteins are considered the ultimate effectors for longevity phenotype, besides genes and transcription factors associated with genetic factors 7, 8. Metabolites, such as fatty acids, amino acids and vitamins, perform diverse functions including energy metabolism and signaling. Proteins, as the products of gene transcription and translation, affect physiological functions by different protein forms, such as cell membrane structure and enzymes. Moreover, post-translational modifications of proteins also have a significant impact on molecular functions. Hence, metabolites and proteins uncovered through investigations of centenarians and their offspring can be regarded as potential longevity-related biomarkers.

Metabolomics and proteomics, as advanced high-throughput omics techniques, are considered the most promising tools for identifying biomarkers 9-12. Metabolomics typically includes untargeted and targeted metabolomics, which is widely utilized to identify metabolites produced in response to all kinds of challenges 13. Proteomics mainly includes both general and post-translational modification (PTM) proteomics, which is applicable to identify proteins regulated by genetic and external stimuli 14. Furthermore, the multi-omics integration of metabolomics and proteomics with genomics, transcriptomics, or microbiomics will provide more precise longevity-related biomarkers in different biomolecular levels. Here, we briefly reviewed longevity-related biomarkers, focusing on metabolomics, proteomics and multi-omics in long-live populations. Additionally, we also discuss the molecular mechanisms that underlying these biomarkers and their role in promoting longevity. We hope to deepen our understanding of the mechanisms behind biomarkers influencing individual aging, providing a theoretical basis to achieve longevity.

## 2. Metabolomics studies associated with longevity

**Figure 1:**
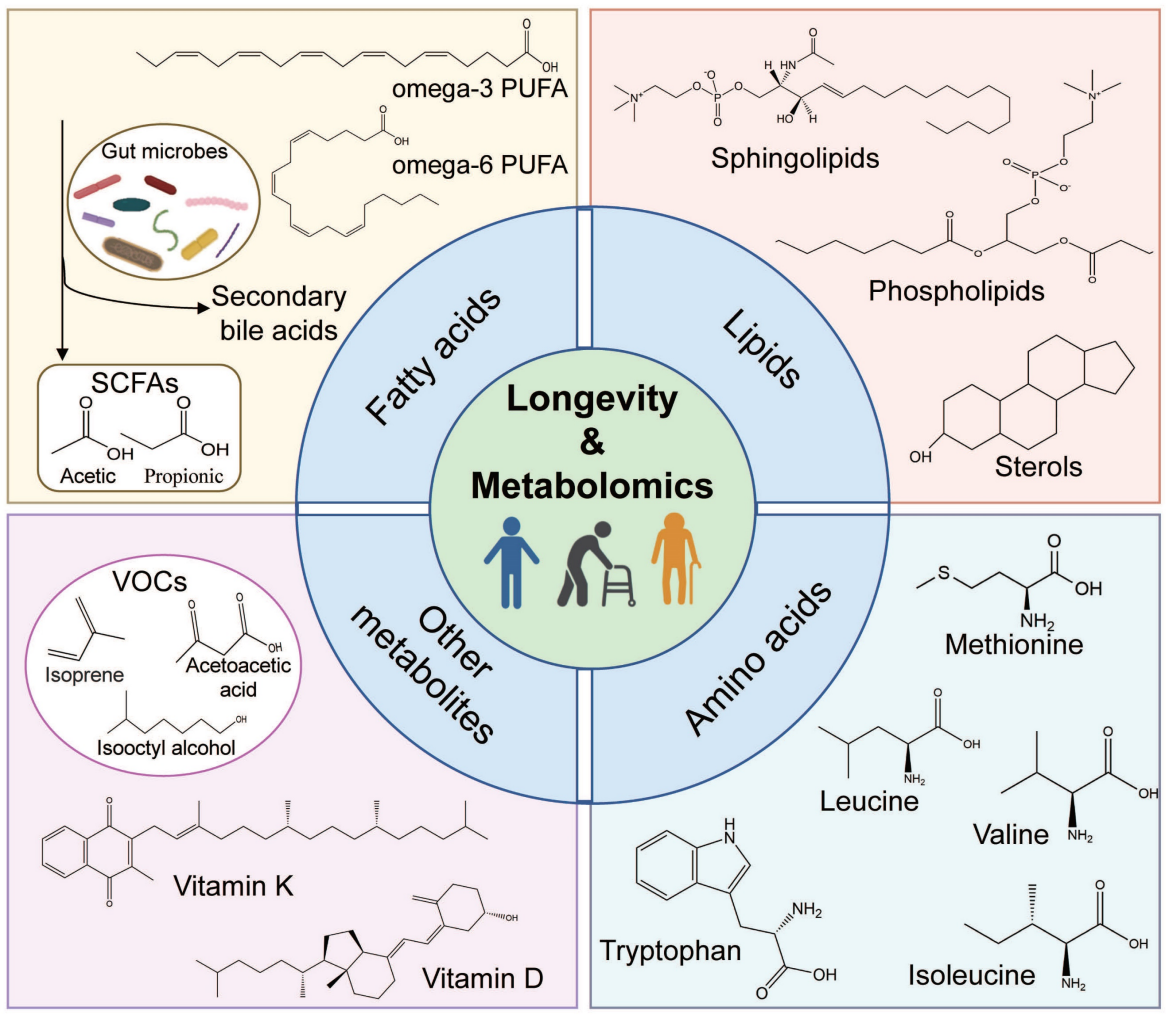
** Schematic representation of longevity-related biomarkers.** The schematic diagram shows the following four main longevity-related biomarkers: fatty acids, lipids, amino acids, and other metabolites. (1) fatty acids: omega-3/6 polyunsaturated fatty acids (PUFAs), secondary bile acids, and short-chain fatty acids (SCFAs, which are derived by gut bacteria utilizing vegetables and fruits); (2) lipids: phospholipids, sphingolipids, and sterols; (3) amino acids: essential and non-essential amino acids, including the branched-chain amino acids (BCAAs); (4) other metabolites: volatile organic compounds (VOCs) and vitamin (such as vitamin D/K).

Metabolomics comprises untargeted metabolomics, involving the universal detection of small molecules, and targeted metabolomics, which selectively detects specific metabolites like lipids or amino acids 15, 16. In this section, we briefly describe the longevity-related biomarkers discovered by metabolomics, including fatty acids, lipids, amino acids, and other metabolites (Figure 1).

### 2.1 Fatty acids

Fatty acids are classified as unsaturated fatty acids and saturated fatty acids. Unsaturated fatty acids, particularly monounsaturated fatty acids (MUFAs) and polyunsaturated fatty acids (PUFAs), have gradually become a focus of longevity research due to their susceptibility to lipid peroxidation compared to saturated fatty acids. Furthermore, gut-bacteria-derived fatty acids, such as bile acids and short-chain fatty acids, also frequently have been drawn focus in longevity studies. In this section, we will focus on the impact of PUFAs and gut-bacteria-derived fatty acids in long-lived populations.

#### 2.1.1 PUFA

PUFAs can be designated as omega-3 PUFAs and omega-6 PUFAs. Eicosapentaenoic acid (EPA) and docosahexaenoic acid (DHA) in omega-3 PUFA family, and arachidonic acid (AA) in omega-6 PUFA family played critical roles in maintaining the integrity of cell structures, producing signaling molecules, and regulating a variety of biological pathways 17.

In the omega-3 PUFA family, EPA and DHA frequently appeared in the study of healthy aging and longevity, which are famous for their potential benefits in reducing triglyceride levels and exerting anti-inflammatory effects 18. EPA and DHA have been reported to be beneficial in preventing cardiovascular disease, cancer, and diabetes in the elderly 19. Xyda *et al.* 20 found that plasma levels of diacylglycerols, phospholipids, and triacylglycerols (TG) decreased dramatically in the elderly taking EPA and DHA supplements. The possible effects of EPA and DHA supplementation were the reduction in total TG, representing a decrease in very low-density lipoprotein (VLDL) particles, and a modest increase in high-density lipoprotein (HDL) cholesterol.

As a member of omega-6 PUFA, AA appeared a lot of attention in longevity studies. Among the metabolites of AA, the serum levels of 8(9)-epoxyeicosatrienoic acid [8(9)-EET] was reported to elevate considerably in centenarians, as a possible result of activation of cell detoxification processes. What's more, the serum levels of 11,12-dihydroxyeicosatrienoic acid (11,12-DHET), linoleic acid, 9-hydroxyoctadecadienoic acid (9-HODE), and 9-oxo-hydroxyoctadecadienoic acid (9-oxo-HODE) were lower in centenarians, which might be a phenomenon of good antioxidant responses 21. Moreover, the EPA/AA ratio seems to have important physiological implications for healthy aging rather than a single EPA level. In the Longevity Sciences-Longitudinal Study of Aging, greater EPA/AA ratios were inversely linked with all-cause mortality, whereas higher serum EPA levels alone were not substantially negatively correlated with mortality 22. The EPA/AA ratio might be a protective factor for longevity through influences on membrane properties, cell signaling, and reduced cardiac events 23. Furthermore, the EPA/AA ratio was highest in healthy centenarians, compared to healthy controls and acute myocardial infarction controls 24. Hence, the EPA/AA ratio could be a potential predictor against major age-related disorders.

**Figure 2:**
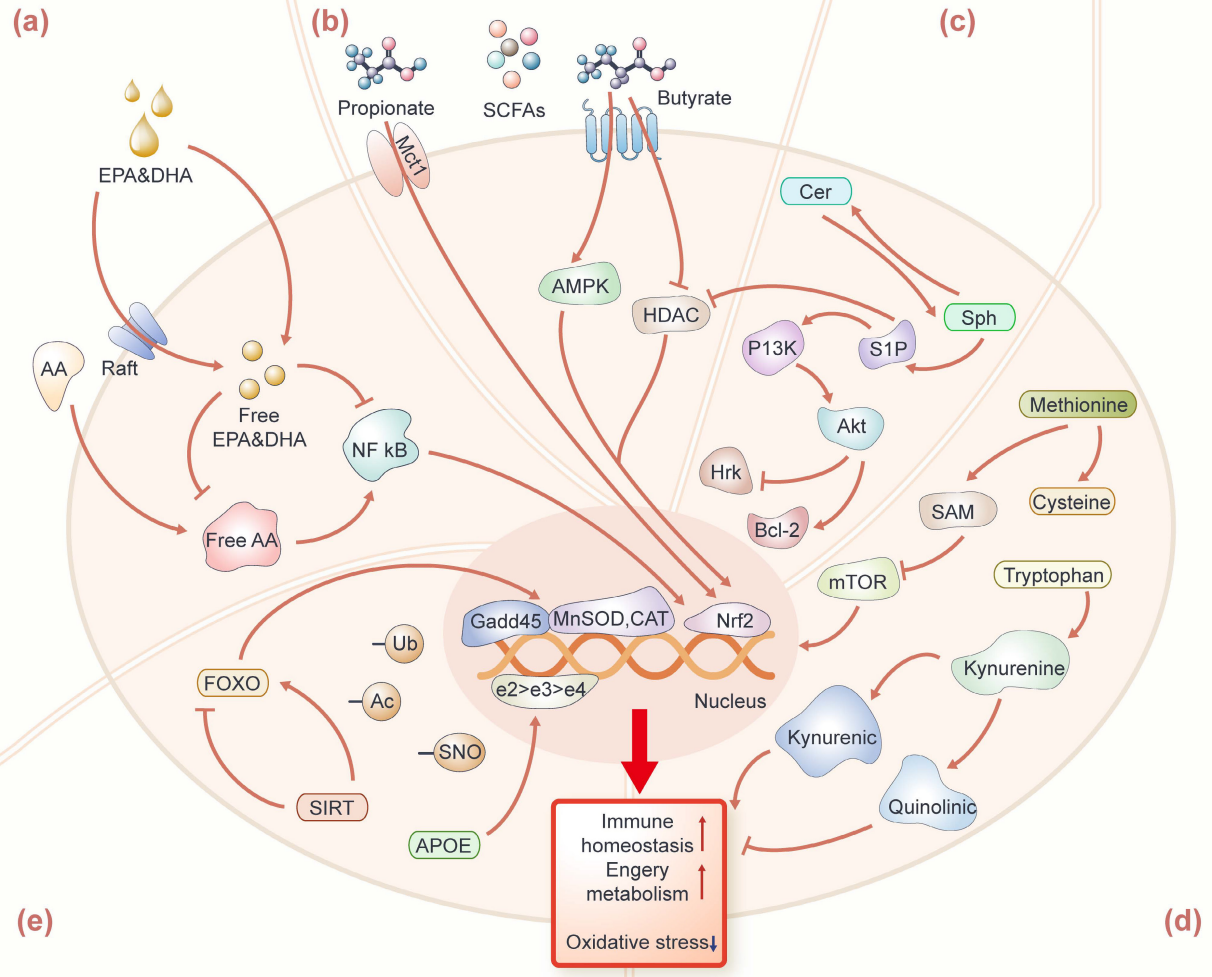
** Mechanisms of longevity-related metabolites and proteins for potentially fostering healthy longevity.** (a) EPA, DHA, and AA are resistant to oxidative stress and inflammation mainly by maintaining the fluidity of the cell membrane, promoting lipid raft assembly, and further inhibiting the activation of NF-κB, and Nrf2 pathways in the cell nucleus. (b) Propionate binds to FFAR3; butyrate inhibits HDAC and promotes the expression of Mct1, all of which further mediate the Nrf2 pathway to maintain immune homeostasis. (c) S1P can reduce the gene expression of pro-apoptotic Hrk protein through the PI3K-Akt pathway and upregulate the expression of the Bcl-2 family to counteract the pro-apoptotic effect of Cer. (d) Methionine enters the methionine metabolism by being converted into SAM, and further inhibiting the mTOR signaling activation. Tryptophan produces kynurenic acid with neuroprotection through the kynurenine metabolism pathway. (e) APOE, FOXO, and SIRT regulate autophagy, DNA repair, energy metabolism, and immune homeostasis by affecting the gene expression of Gadd45, MnSOD, and CAT. Protein modifications include S-nitrosation (SNO), acetylation (Ac) and ubiquitination (Ub), which play an important role in the modification of longevity-related proteins.

As to why EPA and DHA can affect human longevity, scientists have the following conjecture that it is generally believed that they have the regulation of the immune function on cells. The impact of EPA, DHA, AA, and their metabolites in omega-3 or 6 fatty acids are the regulation of the immune function on cells. EPA and DHA, acting as activators for anti-inflammatory transcription factors 25, 26, can compete with AA to bind to substrates in the enzymatic pathways catalyzed by cyclooxygenase (COX) and lipoxygenase (LOX), thereby inhibiting the conversion of AA into pro-inflammatory molecules such as prostaglandins and leukotrienes (Figure 2a). Studies suggest that the antioxidant properties of EPA and DHA are mediated by activating nuclear factor erythroid 2-related factor 2 (Nrf2), involving the upregulation of hemoglobin oxygenase 1 (HO-1) to protect the brain from ischemic damage 27. Therefore, effective inducers of Nrf2 activation can be considered as an effective measure to prevent inflammation-mediated diseases. Simultaneously, the increasing EPA and DHA levels in circulation facilitate the aggregation of signaling membrane proteins and lipids to form lipid rafts 28, 29, and then reduces the transmission efficiency of cellular inflammatory signals and the activation of pro-inflammatory transcription factors such as Nuclear factor kappa B (NF-κB) 26. Additionally, omega-3 or 6 fatty acids can regulate the balance of gut microbes, enhancing fatty acids absorption, utilization, and biotransformation 30, 31. In conclusion, changes in human PUFAs (e.g., EPA, DHA, and AA) and the ratio of EPA/AA may be potential biomarkers of longevity.

#### 2.1.2 Gut-bacteria-derived fatty acids

Gut bacteria have been proven to involve human health and impact longevity by fatty acid metabolic pathway 32-34. Of these, bile acids and short-chain fatty acids (SCFAs) are famous metabolites derived from gut bacteria. Bile acids include primary and secondary bile acids, and the latter are the products formed by decomposition of primary bile acids by gut bacteria. Regarded bile acid metabolites, the levels of those (such as iso-, 3-oxo-and isoallo-lithocholic acid (LCA)) generated from *Odoribacteraceae* in centenarians' feces were significantly increased, and relatively low in the elderly and young controls 33. The unique biosynthetic pathway for isoallo-LCA is that 3-oxo-Δ^4^-LCA conversion to isoallo-LCA is mediated by 5AR (3-oxo-5-alpha-steroid 4-dehydrogenase) and 3β-HSDH (short-chain dehydrogenase). Subsequently, *Odoribacteraceae* St21 was administered to mice infected with *Clostridioides difficile* (Gram-positive pathogens), resulting in reduced intestinal *C. difficile* numbers and enhanced production of isoalloLCA to defend against infection, a mechanism that could potentially delay aging. Furthermore, LCA also were enriched in nonagenarians and centenarians' plasma, which can be detoxified to form lithocholic acid sulfate via gut bacteria such as *Aeromonas veronii* and *Butyrivibrio crossotus* 35. Overall, gut microbe alterations and bile acid metabolic differences will affect gut homeostasis.

SCFAs are the major products of dietary fiber fermentation by gut bacteria. The prevalent SCFAs in humans (76-95 yrs) are mainly acetic acid, propionic acid, and butyric acid 36. The levels of these prevalent SCFAs in fecal were decreased in the elderly (>80 yrs), compared with young adults (50-80 yrs) 37. In nonagenarians and centenarians, these prevalent SCFAs mentioned above were over-represented with respect to their children (50-79 yrs), which were produced by *Parabacteroides* and further affected gut homeostasis 35. In addition, branched SCFAs such as isobutyric and isovaleric were elevated in centenarians 33. Existing studies have elucidated the molecular mechanisms through which SCFAs sustain human health, predominantly by modulating the integrity of cellular barriers, such as the intestinal barrier and the blood-brain barrier (Figure 2b). Among these, propionate and butyrate positively influence longevity by promoting the repair and regeneration of intestinal cells, maintaining intestinal mucosa integrity, and regulating the activity of the immune system 38. Propionate can bind to the free fatty acid receptor 3 (FFAR 3) in the human brain endothelial cells and then protect the blood-brain barrier from oxidative stress through the Nrf2 pathway 39. Butyrate can mediate protection against endothelial dysfunction in mice by inhibiting protein deacetyase (HDAC) -mediated activation of Nrf2 40. The newest article found that butyrate can also alleviate the imbalance of muscle satellite cell homeostasis caused by antibiotic-induced intestinal microflora disorders, and promote the expression of monocarboxylate transporter 1 (Mct1) to regulate muscle satellite cell homeostasis 41. Thus, propionate and butyrate are potential biomarkers for centenarians to achieve health and longevity, and exploring more other SCFAs derived from salutary microorganisms could be beneficial to the discovery of novel longevity-related biomarkers.

Apart from the fatty acids discussed above, some intermediate products in fatty acid oxidation also play important roles in health and longevity, such as β-hydroxybutyrate 42. Studies have shown that mice fed a high-fat diet and supplemented with the ketone body β-hydroxybutyrate displayed improvements in metabolic health and memory. These results suggest that dietary supplementation with β-hydroxybutyrate may prove to be an effective intervention for the future treatment of age-related dysfunction and further promote healthy longevity 43. Therefore, the downstream products of fatty acids are also worthy of attention in promoting healthy longevity.

### 2.2 Lipids

Lipids are a broad group of organic compounds which include glycerol, fatty acids, phospholipids, sterols, and others. They exhibit diverse structures, often comprising one or more fatty acid molecules bound to other compounds. Other lipids besides fatty acids have also played an important role in longevity. This part primarily discusses lipids other than fatty acids. Lipidomics is one of the targeted metabolomics approaches, which enables the extraction and optimization of lipid molecule separation based on their lipophilic properties. Compared to untargeted metabolomics, lipidomics mainly provides a more precise detection method for some specific lipid molecules (such as phospholipids, sphingolipids, sterols, etc.) (Table 1).

Phospholipids, sphingolipids, and sterols are common types of lipids. (1) Phospholipids include phosphatidylcholine (PC), phosphatidylethanolamine (PE), ether phosphatidylcholine (PC-O), lysophosphatidylcholine (LPC), phosphatidylcholine (LPE), which maintain membrane fluidity. (2) Sphingolipids, such as sphingomyelins (SMs) and ceramides (Cers), are ubiquitous building blocks of cell membranes, which are involved in cell biological processes 44. (3) Sterols play crucial roles in both cell proliferation as essential components and as significant signaling molecules, including cholesterol, steroids, estrogen and cholesterol ester (CEs) 45. The species and molecular composition of lipids influence cellular distribution, metabolism and subsequently impact on cellular aging.

The abundance of lipids species in centenarians is determined by the number of carbon atoms and double bonds of lipids species. Pradas *et al.* analyzed plasma ether lipid profiles of centenarians, elderly and young in Spain, and identified lipids phenotype with exceptional human longevity 46. The results revealed that fifteen LPC lipid species, such as LPC(O-24:0) and LPC(O-24:1) with lower carbon atomic numbers and double bonds in centenarians were significantly increased and predominated the presence in alkyl form. Jové's team observed an increase in the levels of phospholipids—including PS (40:3), PC (40:5), PE (33:3 34:2), LPC (18:1), LPE (24:1), and CEs—among centenarians and adults compared to other groups 47. Similarly, the concentration of most unsaturated diacyl PC and LPC species rose significantly among centenarians and adults 21. Collino *et al.* found that the concentrations of tiny PC, LPC, and PC-O remained unchanged until age 70 and underwent significant changes with longer carbon chains and double bond numbers in centenarians compared to the elderly and the young 48. It is unclear whether these compositional differences are benefits or drawbacks for longevity.

Nevertheless, it can be inferred that the lipid species composition in centenarians has undergone remodeling to enhance resistance against lipid peroxidation. For example, serum PC-O species are positively correlated to the HDL circulating level, supporting their role as antioxidants preventing lipoprotein oxidation 21, 46.

Some sphingolipids in long-lived individuals have also attracted the attention of scientists. Sphingolipids are categorized as structural or signal sphingolipids based on location and function. Structural sphingolipids are found primarily in cell membranes, including the myelin sheath of neurons, where they provide support and protection to the cells. Signal sphingolipids involve functions in the process of cell signal transmission. A recent study based on targeted sphingolipidomic in plasma found that centenarians have a significant increase in sphingolipid species, such as monohexosylceramides (HexCer), trihexosylceramides, and gangliosides (GM), which have structural roles 49. Barbacini *et al.* also found similar levels of sphingolipids in the serum of centenarians (increased in HexCer and GM) 50. Sphingosines, sphingosine-1-phosphate (S1P), ceramide-1-phosphate, and Cers with signaling roles remained unaltered among cohorts. Additionally, very long-chain ceramides like C24:0 and the ratio to the long-chain ceramide C16:0 (i.e., C24:0/C16:0) have been found to be associated with better cardiovascular health, lower cardiovascular event rates as well as lower all-cause mortality 51. Therefore, structural glycosphingolipids with signaling roles may be key factors in attaining longevity. Some studies have found that the increase of S1P and the decrease of Cers are conducive to neuronal stress resistance (Figure 2c). S1P can counteract the pro-apoptotic effects of ceramides by reducing oxidative stress, decreasing the gene expression of pro-apoptotic Harakiri (Hrk) protein, and upregulating the expression of the B-cell lymphoma 2 (Bcl-2) family of pro-apoptotic proteins 52, which appear to influence cell metabolism, stress response, and immune status through the PI3K/Akt pathway 53, 54. Enzymes involved in sphingolipid metabolism in centenarians such as sphingomyelin phosphodiesterase 3 (SMPD3) have been verified to be over-expressed at PBMC mRNA and protein levels. This study revealed that the synthesis of Cers in centenarians is obtained by SM degradation. Overall, targeted sphingolipidomic profiling of centenarians enhances our understanding of longevity mechanisms that warrant further investigation.

Since sterols are related to hormones, the study of the large cohorts has proven that sterols are affected by sex 55. Sterols play an important role in the gender differences in longevity among centenarians. Studies have shown that estrogen can influence the immune and antioxidant systems, as well as help maintain telomere length 56-58. Baseline data from the China Hainan Centenarian Cohort Study indicated that compared to elderly women in the perimenopausal and postmenopausal stages, female centenarians have a non-protective association between estradiol and progesterone levels above a certain threshold and all-cause mortality 59. Therefore, in lipidomics studies of longevity cohorts, it is necessary to further evaluate hormone levels related to gender.

### 2.3 Amino acid metabolites

Amino acids are important signaling molecules to regulate the physiological processes in humans. Essential amino acids such as methionine and tryptophan, along with non-essential amino acids like branched-chain amino acids (BCAAs), have garnered attention in the study of longevity.

Methionine is an essential amino acid that the body cannot generate, and is one of the four common sulfur-containing amino acids (methionine, cysteine, homocysteine, and taurine). Methionine metabolism pathway has been proven to upregulate in long-lived individuals 60, 61, which involved various biochemical reactions, such as transmethylation and transsulfuration 62. Methionine enters the methionine metabolism by being converted into S-adenosylmethionine (SAM), a universal donor for methyl transfer reactions, thus participating in the methylation process of a variety of biological molecules including DNA, RNA and proteins 63, hence SAM plays a crucial role in maintaining vital biological processes in the elderly (Figure 2d). SAM is converted to homocysteine under the action of glycine N-methyltransferase (Gnmt) and S-adenosylhomocysteine hydrolase (AHCY), and then homocysteine is converted back to methionine under the action of methionine synthase, completing the cycle of methionine. Here, we observed a unique pattern of methionine metabolism in the blood circulation of centenarians compared to other controls. Plasma methionine in centenarians did not differ between elderly and young, while methionine metabolites (such as cystathionine, cysteine and taurine) were significantly higher than the other two groups, while the homocysteine levels of centenarians were significantly lower than the other two groups, which may be beneficial to maintaining healthy lifespan and reducing the risk of chronic diseases in centenarians 61. Understanding the unique patterns of methionine metabolism observed in centenarians prompts further investigation into its implications across different species, particularly for mice as the model animal. Methionine restriction in mice was found to reduce serum IGF-I, insulin, glucose, thyroid hormone and inflammation levels, and increase MIF (macrophage migration inhibition factor) levels in hepatocytes, leading to resistance to multiple diseases and slower aging 64. Homocysteine can increase the level of oxidative stress and activate the TOR signaling pathway, thereby accelerating the aging process 65, 66. Thus, methionine restriction and lowering homocysteine levels contribute to maintaining the best physiological state in centenarians. Finally, methionine intake may be decreased by vegetarian diets, which possibly promoted health benefits 67, and the dietary habits of centenarians are also worth further investigating.

Tryptophan, an essential amino acid, as the precursor of the neurotransmitters-serotonin and melatonin, plays a crucial role in human health 68. In a plasma metabolomics study, examining centenarians, nonagenarians, elderly in longevity region and controls in non-longevity region, the results indicated that the tryptophan biosynthesis pathways were significantly enriched in centenarians 69. Mota-martorell *et al.* discovered decreased levels of tryptophan in centenarians' plasma 61, and this result is consistent with previous studies of declining tryptophan levels in centenarians 21. Additionally, tryptophan was also decreased in centenarians' urine compared to the elderly 70. As previously reported, a low level of tryptophan is considered to be related to the rise of chronic low-grade inflammatory disorders and immune activation in centenarians 71, 72. Tryptophan can be metabolized to kynurenine, which further produces several metabolites, including kynurenic acid and quinolinic acid. It has been reported that frail elderly have lower kynurenic acid compared to quinolinic acid 73. Studies have shown that kynurenic acid increases energy utilization by activating G protein-coupled receptor Gpr35 74 (Figure 2d). The elevation of kynurenic acid and Gpr35 enhance cellular respiration, and increase the levels of Rgs14 in adipocytes, which leads to enhanced energy homeostasis. Quinolinic acid has been linked to neurodegenerative diseases and has been shown to impair mitophagy, thereby promoting microglial senescence, thereby contributing to the aging process of the brain 75. Thus, the tryptophan and its metabolism play a crucial role in maintaining health status in centenarians.

The catabolism of branched-chain amino acids (such as valine, leucine, and isoleucine) is reported to be a conserved regulator of physiological aging, which can promote aging though activating the target of rapamycin/ribosomal protein S6 kinase (TOR/S6K) signaling and the target of rapamycin complex 1 (TORC1) pathway 76, 77. In a study based on a longevity cohort in Guangxi, China 78, the oldest-old group always had relatively low branched-chain amino acid levels. In centenarians, lower valine levels were observed with respect to adults and aged individuals 61. Studies have found that high levels of BCAAs in the blood are associated with an increased risk of cardiovascular diseases and neurodegenerative diseases. A Mendelian randomization study using data from the UK Biobank and other databases found that individuals genetically predisposed to higher circulating levels of BCAAs had a greater risk of peripheral arterial disease and stroke 79, 80. Moreover, there is evidence from a bidirectional Mendelian randomization study that points to Alzheimer's disease being associated with decreased BCAAs levels 81, while leucine has been demonstrated to promote AD via a mTOR-dependent mechanism 82. Furthermore, the restriction of isoleucine has been shown to effectively reduce obesity and improve glucose tolerance, reversing the impact of age on several molecular markers of aging such as phosphatidylglycerol, promoting healthy lifespan and extending longevity in young or adult mice 83. In other report, isoleucine restriction also promotes upregulation of fatty acid metabolism and downregulation of immune pathways in male mice, suggesting potential effects of isoleucine restriction on metabolism and inflammation in healthy aging 84. In conclusion, lower levels of these amino acids may contribute to longevity, and the underlying mechanisms should be further studied.

### 2.4 Other metabolites in longevity associated study

As far as other metabolites, numerous noteworthy molecules are concerned, including vitamins and volatile organic compounds (VOCs). Vitamin D is a secosteroid (pro)-hormone that is well recognized to regulate bone metabolism, and vitamin D deficiency is frequently presented in elderly 85. High vitamin D level is associated with better cognitive function in Ashkenazi Jewish centenarians 86. The study of the Chinese centenarians' cohort has observed that vitamin D deficiency is a predictor of functional dependence 87. Thus, centenarians have higher blood Vitamin D levels, which may explain their excellent health and exceptional lifespan. Vitamin K is also known as the clotting vitamin, which exists in the form of phylloquinone and menaquinones. Recently, a study described that menaquinones-4 was the predominant vitamin in brain samples from 48 decedents (aged 98-107) enrolled in the Georgia Centenarian Study 88. Furthermore, circulating phylloquinone were significantly higher in nondemented centenarians than in demented, which might reflect intake of vitamin K-rich foods.

VOCs have been discovered by metabolomics based on GC-MS 89. VOCs are low-weight carbon-based molecules that reflect the metabolic conditions of the individual, affected by age, gender, diet, physiological and habits. Therefore, VOCs could be considered as “odor-fingerprint” of individuals 90 and non-invasive diagnostic biomarkers 91-93. Conte M *et al.* investigated the profile of VOCs in both urine and feces samples from 73 volunteers, including centenarians 94. Surprisingly, the profile of VOCs can separate for the couples “centenarians-offspring” or the trios “centenarians-offspring-spouse”. Octanoic acid, ethanol, and phenol 4methyl can explain the majority of similarity between the couple centenarian-offspring, while cyclopentanol, benzaldeide, and acetic acid butyl ester can explain the majority of similarity between family trios (centenarian, offspring and spouse). Overall, the above metabolites are worth exploring to provide new insight to understand the mechanism of longevity.

## 3. Proteomics studies associated with longevity

**Figure 3:**
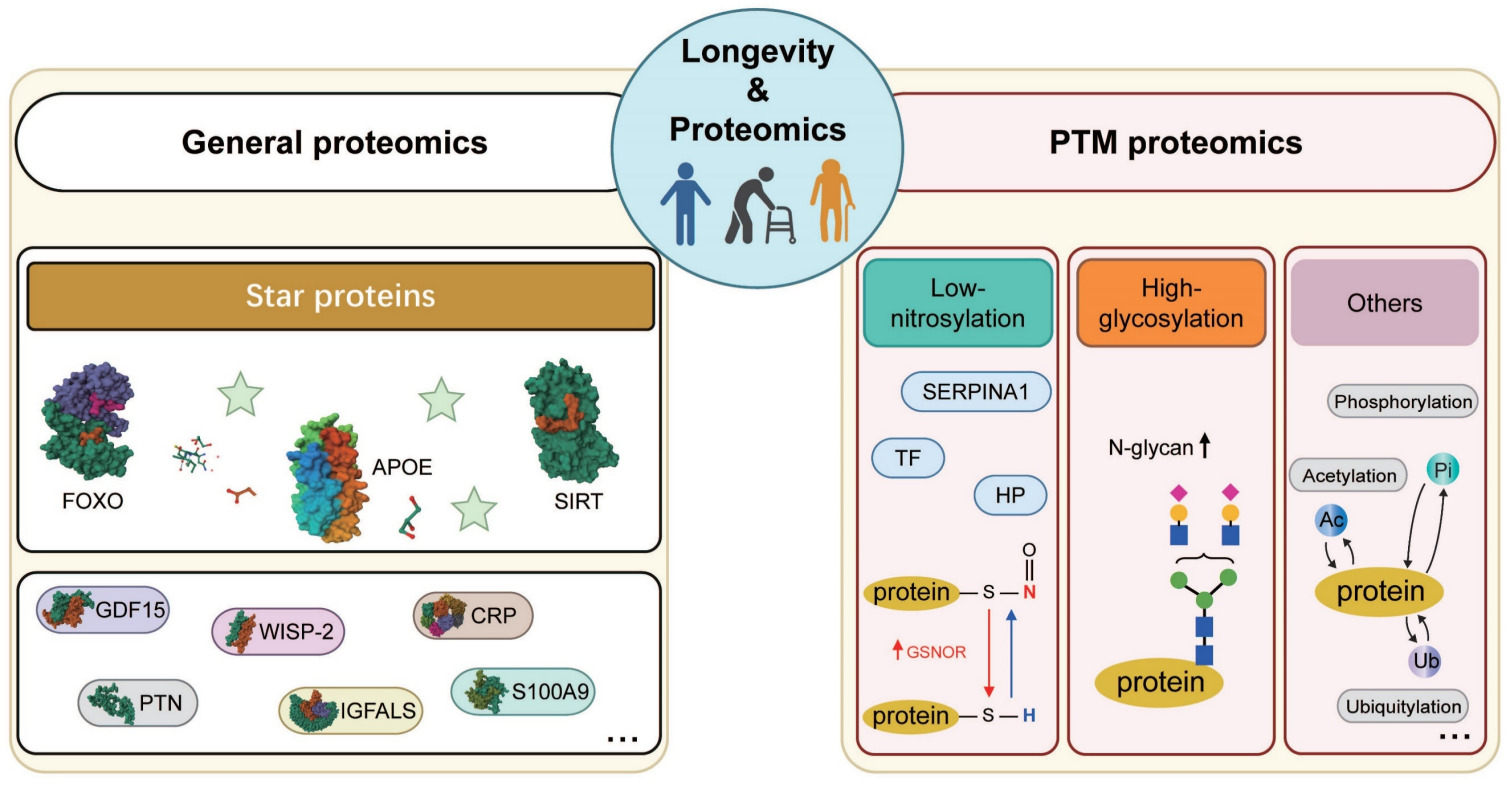
** Schematic representation of longevity-related biomarkers.** Schematic depictions of major longevity-related biomarkers identified from both general and post-translational modification (PTM) proteomics. (1) Longevity-related biomarkers identified from general proteomics: apolipoprotein E (APOE), forkhead box gene, group O proteins (FOXO), sirtuin proteins (SIRT). Besides, there are other longevity-related protein biomarkers repeatedly identified from general proteomics in different studies, such as GDF15, PTN, SFRP1, WISP-2, IGFALS, and SERPINF2. (2) Longevity-related biomarkers identified from PTM proteomics: low-nitrosylated related proteins (such as TF, HP, and SERPINA1) and high-denitrosylated related proteins (GSNOR), as well as high N-glycan related proteins. These longevity-related proteins mentioned above are identified by nitrosoproteomics and glycoproteomics. In addition, other longevity-related biomarkers are expected to be obtained through the following PTM proteomics, such as phospho-proteomics, acetyl-proteomics, and ubiquitin-proteomics. Abbreviations: PTN: Pleiotrophin; protein 2; IGFALS: Insulin-like growth factor-binding protein complex acid labile subunit; SERPINF2: Alpha-2-antiplasmin; TF: Serotransferrin, HP: Haptoglobin, SERPINA1: α1-antitrypsin; GSNOR: nitrosoglutathione reductase. The structure of N-glycan is cited in the paper (Miura, Yuri *et al.*, 2015). N-glycan: blue square, N-acetylglucosamine; yellow circle, galactose; green circle, mannose; purple diamond, N-acetylneuraminic acid.

Proteomics encompasses both general proteomics, which quantitatively profiles various proteins, and post-translational modification (PTM) proteomics, which identifies chemical modifications and specific sites on polypeptide chains, such as nitrosylation and glycosylation 95, 96. In this section, we will briefly describe longevity-related biomarkers discovered by general proteomics, including Apolipoprotein E (APOE), Forkhead box O (FOXO), and sirtuin (SIRT), which have been noticed by scientists since the 1990s 97, 98, and other vital proteins (Table 2). Moreover, PTM proteomics revealed post-translational modifications of longevity-related proteins, such as lower nitrosylation and higher N-glycan, which can further provide us with a deeper insight into longevity (Figure 3).

### 3.1 Longevity-related biomarkers based on general proteomics

#### 3.1.1 "Star proteins"

In recent decades, scientists have successively discovered several longevity-related proteins through experiments involving nematode worms, mouse models, and population surveys, elucidating the mechanisms governing lifespan 99-101. These proteins, which we refer to here as "star proteins", include APOE 102-104, FOXO 8, 105-108, and SIRT 100, 109, 110.

APOE is a transport protein found in plasma and interstitial fluid and the *APOE* gene has three common alleles: e2, e3, and e4, which play a crucial role in lipid metabolism, cognitive function, and immune regulation (Figure 2e). APOE is indispensable in lipid metabolism and can assist in the distribution of triglyceride-rich lipoproteins to various tissues and cells 111. Gene *APOE* in different alleles may lead to different changes in cholesterol metabolism. The association between *APOE* gene loci and lifespan has been confirmed by various studies based on genome-wide association studies 112-116 and centenarians have a higher frequency of the *APOE* e2 allele. The e2 allele have a neuroprotective effect 102, 117, 118, while the e4 allele is recognized as a risk factor for Alzheimer's disease and impaired cognitive function 119-121. Sebastiani *et al.* utilized the expression levels of e2, e3, and e4 alleles to correlate with serum proteins in serum proteomics targeting centenarians and their offspring 122. The study indicated that the prevalence of e2 allele was higher in centenarians and offspring compared to controls. Studies have shown that centenarians with the *APOE* e2 allele have lower total cholesterol and LDL levels, and higher HDL and TG levels in plasma 123. Building upon this foundation, Sebastiani's team conducted a meta-analysis by integrating data from five longevity cohorts on the population prevalence of *APOE* alleles e2 to e4 and metabolomics data. They revealed that the e2 allele might be related to changes in levels of lipid-bound polyunsaturated fatty acids, as well as with the prevalence of beneficial gut bacteria such as *Akkermansia* and* Lachnoclostridium*, thereby affecting lipid metabolism conducive to healthy longevity 124. In conclusion, proteins associated with the *APOE* e2 allele appear to have a positive impact on healthy longevity, while proteins associated with the e4 allele might exert negative effects. They revealed that the e2 allele might be as well as with the prevalence of beneficial gut bacteria such as *Akkermansia* and *Lachnoclostridium.*

FOXO and SIRT are considered prominent proteins associated with longevity. FOXO can bind to specific DNA sequences, playing a crucial role in regulating gene expression related to cell growth, differentiation, metabolism and autophagy 99. The polymorphism of the FOXO gene has been found to be closely linked to longevity in different populations from various regions, such as the Japanese (FOXO3A rs2764264, rs13217795 and rs2802292) 105, the Chinese (FOXO3 rs10499051, rs7762395, rs4946933 and rs3800230) 125, and the American (FOXO3 rs6911407 and rs2253310) 108. Studies have shown that the copy number of the rs2802292 G allele at the FOXO3 locus is associated with a reduced incidence of age-related diseases in centenarians, and its importance in promoting reactive oxygen species (ROS) detoxification, redox balance and DNA repair was found in the human HAP1 homologous cell line (G/T) 126. Similarly, long-lived individuals carrying the rs2802292 G allele had lower plasma TNF-α than non-carriers, suggesting that FOXO3 may be involved in oxidative stress 127. Studies have found that FOXO3 may be essential for the lifespan-extending effects of dietary restriction (DR), as WT-DR mice live longer compared to *foxo3*^+/-^ or *foxo3*^-/-^-DR mice 128. In addition, FOXO3 also contributes to mitigating intestinal neurodegeneration associated with inflammation 129. It maintains cellular homeostasis by promoting the transcription of antioxidant genes such as manganese superoxide dismutase (MnSOD) and catalase 105, 130, 131, while also regulate growth arrest DNA damage-inducible 45 (GADD45) and DNA damage response genes to resist stress 132 (Figure 2e).

SIRT is a family of nicotinamide adenosine dinucleotide (NAD) dependent histone deacetylases and adenosine diphosphate (ADP) ribose transferases 133. SIRT can interact with the FOXO family to better respond to cellular oxidative stress 134-136. Interestingly, genetic polymorphisms in the SIRT3 and SIRT6 genes have been found to be closely associated with human longevity 137-139, in which SIRT6 has been proven to be related to enhance neuroplasticity and improve cognitive function 140. Studies have shown that brain-specific Sirt1-overexpressing (BRASTO) transgenic mice exhibit significant lifespan extension in both males and females 141. Aged BRASTO mice demonstrate phenotypes consistent with delayed aging: they exhibit significant enhancements in physical activity, body temperature, and quality of sleep, compared to age-matched control mice. Additionally, mitochondria morphology and function of skeletal muscle appear younger. This is attributed to Sirt1 enhancing Ox2r promoter activity through Nkx2-1 deacetylation to regulate bodily functions. Moreover, FOXO and SIRT regulate autophagy genes expression, which can promote the degradation of harmful proteins and alleviate oxidative stress 142, 143. Knockdown of SIRT4 gene accelerated cell senescence and led to an increase in the senescence-associated secretory phenotype (SASP), a finding that suggests SIRT4 as a potential target for extending lifespan 144. On the other hand, they regulate insulin signaling pathways and metabolic enzymes expression to prevent age-related metabolic diseases 145. FOXO and SIRT can also reduce inflammation by inhibiting NF-κB pathway 146. To sum up the aforementioned findings, FOXO and SIRT are potential targets in the regulation of longevity, although their roles in the proteomics of long-lived populations have not been reported thus far.

#### 3.1.2 Other vital proteins

Nowadays, general proteomics is utilized to identify other proteins associated with extreme longevity in large-scale longevity research cohorts, such as Pleiotrophin (PTN), WNT1-Inducible Signaling Pathway Protein 2 (WISP-2), and Growth/Differentiation Factor 15 (GDF15), Insulin-like Growth Factor-Binding Protein Complex Acid Labile Subunit (IGFALS).

PTN, WISP-2, and GDF15 have been identified as potential longevity-related biomarkers. Sebastiani *et al.* analyzed plasma protein profiles of the New England Centenarian Study cohort by SomaLogic technology, in which PTN and WISP-2 were found to increase with age and were highly elevated in centenarians compared to control groups 147. Elevated levels of PTN have also been positively correlated with healthy aging in the previous cohort studies 148-150. In long-lived offspring, WISP-2 levels increase with age, especially in females 151. WISP-2 inhibits cardiac hypertrophy and fibrosis 152 and promotes the survival of vascular smooth muscle cells 153-155. Thus, upregulated WISP-2 could be speculated to foster cell growth and proliferation, further exerting a positive influence on longevity. Additionally, Sebastiani *et al.* found lower levels of GDF15 expression in individuals with an expected lifespan exceeding 11 years, compared to those with an expected lifespan of less than 10 years, which implied that lower GDF15 levels had a favorable link to an extended lifespan 147. High levels of GDF15 are negatively correlated with muscle strength in the elderly in another research 156. In short, lower levels of GDF15 might be considered characteristic proteins for achieving longevity.

IGFALS was found to significantly elevate in the plasma of centenarians, which was also discovered to be linked to some biological processes like vascular generation and B-cell mediated immune response 157. Another study indicated the connection between IGFALS and longevity though its interaction with Insulin-like Growth Factor Binding Protein 3 (IGFBP3); and IGFBP3 variant (rs11977526) was related to the longevity 158. Additionally, IGFBP-3 is associated with the induction of cellular aging 159, 160, which shows lower levels in the plasma of long-lived individuals (>95 yrs), suggesting a potential mechanism for alleviating cellular aging 161. The significance of IGFALS and IGFBP3 in the context of longevity need further validation by more studies of model animals and cohorts.

Moreover, inflammation-related proteins were found to be down-regulated through proteomics in the long-lived population of the MrOS cohort and the LonGenity cohort, such as TNFR (TNF Receptor), complement C9, C7, S100A9 (S100 calcium binding protein A9), and CRP (C-reactive protein), which are mostly involved in inflammatory or complement activation pathways 151, 162.

### 3.2 Longevity-Related Biomarkers Based on Post-translational modification (PTM) Proteomics

Protein modifications commonly include nitrosylation, glycosylation, phosphorylation, acetylation, and ubiquitination. Lower levels of nitrosylation and higher levels of N-glycosylation are reported to be closely associated with longevity in centenarians 163, 164. Exploring the functions of proteins that undergo various modifications by PTM proteomics enables a deeper comprehension of the molecular mechanisms in proteins.

Nitrosoproteomics investigates the impact of protein nitrosylation on structure and function, aiming to understand the function of nitrosylated protein in cells and organisms 165, 166. During cell senescence, cells were influenced by oxidative stress caused by the excessive accumulation of ROS and nitric oxide (NO), which in turn affects the nitrosylation levels of certain proteins 167, 168. Capitanio *et al.* employed S-nitrosoproteomics to analyze plasma proteins in centenarians and adults 163, revealing reduced S-nitrosylation levels in certain plasma proteins, including transferrin (TF), haptoglobin (HP) and alpha-1-antitrypsin (SERPINA1), among centenarians compared to controls. In other word, lower S-nitrosylation levels in centenarians may indicate a beneficial state of maintaining lower levels of ROS and NO. Furthermore, denitrosylation is the reverse process of S-nitrosylation, which is mediated by S-nitroso glutathione reductase (GSNOR) 169. Due to limitations in techniques, there is currently no corresponding proteomics study reported for denitrosylation. To further investigate the dynamic changes in S-nitrosylation and denitrosylation, Rizza *et al.* found that mRNA levels of GSNOR significantly rised in centenarians and adults compared to the elderly 170. And they further displayed that the absence of GSNOR protein will increase S-nitrosylation levels through animal experiments. In summary, lower S-nitrosylation and higher denitrosylation levels may enhance the ability to cope oxidative stress from cellular aging.

Glycoproteomics enables the assessment of glycoprotein structures, glycosylation sites, and the heterogeneity of glycosylation sites 171. Many researchers discovered glycosylation-related factors associated with aging by glycoproteomics, like IgG N-glycosylation 172, 173, and advanced glycation end products 174. Miura was the first to discover a link between plasma protein N-glycan levels and extreme longevity in long-lived populations 164. Semi-supercentenarians (SSC, >105 yrs) exhibited heightened anti-inflammatory responses characterized by increased multi-branched and highly sialylated N-glycan in plasma proteins, as well as agalacto- and/or bisecting N-glycans, distinguishing from both the elderly and young populations. Subsequently, Miura conducted further analysis of characteristic glycopeptide linked to longevity, uncovering a noteworthy augmentation in tri-antennary and sialylated N-glycans at Asn207 and Asn211 sites in haptoglobin among SSC. This extension further prolonged the half-life of haptoglobin, allowing it to bind to free hemoglobin and act as an antioxidant 175. Therefore, N-glycan with high glycosylated levels serves as a characteristic biomarker of centenarians.

Additionally, there are relatively few research reports in longevity cohorts using proteomics such as phosphorylation, acetylation, and ubiquitination. These methods are primarily employed to study the modifications of proteins associated with diseases. Phosphorylation and acetylation proteomics have been highlighted in Alzheimer's disease 176, 177. Furthermore, the phosphorylation of AMP-activated protein kinase (AMPK) 178, acetylation of FOXO proteins 179, and ubiquitination of ubiquitin ligase CHIP 180 are linked to longevity, impacting protein structural, energy metabolism, and stress response. With technological advancements, these PTM proteomics can be employed to explore biomarkers in longevity cohorts.

## 4. Multi-omics studies associated with longevity

Advancement in omics technology reveals that single omics cannot fully address the objectives of scientific study. Multi-omics, integrating genomics, transcriptomics, proteomics, metabolomics, and microbiomics data, is a comprehensive strategy to investigate the causes of healthy aging and lifespan extension in humans. Recently, several recent studies using multi-omics have made significant progress in elucidating the intricate factors contributing to longevity.

Multi-omics integrated analysis using metabolomics and proteomics has gained considerable traction in various fields, such as cognitive impairment 181, dementia 182, and Alzheimer's disease 183. In a study conducted by Ahadi, longitudinal and deep multi-omics profiling in transcripts, proteins, metabolites, cytokines, and microbes was performed on 106 healthy individuals aged 29 to 75 184. And this study revealed distinct age-associated trends and levels of association for 184 molecules across multi-omics, including *Clostridium cluster IV* Fetuin-B and PROS1. In addition, different types of aging patterns are defined according to the molecules level of changes over 2-3 years, called “ageotypes”. Similarly, Tebani *et al.* also conducted a longitudinal multi-omics study over 2-3 years with a cohort of 100 people, aiming at interpret differences between individuals 185. The findings indicate that the alpha subunit of glycoprotein hormones (CGA) exhibits higher expression in women compared to men. Furthermore, γ-tocopherol has been identified as one of the most stable metabolites in this cohorts. Overall, each population or individual exhibits a distinct molecular profile; thus, centenarians, representing a notably long-lived demographic, merit detailed examination of their unique molecular biomarkers.

Centenarians have younger epigenetic characteristics. At the protein level, centenarians may show more favorable histone modification patterns, such as histone acetylation and methylation, which are often closely related to gene expression regulation 186, 187. At the nucleic acid level, centenarians tend to have lower global DNA methylation levels, which is associated with slower biological aging and longer lifespan 188, 189. For example, both centenarians and their offspring resulted significantly epigenetically younger than the control though epigenetic age measures by DNA methylation values of CpG sites, with mean epigenetic age discrepancy equal to -6.45 and -1.65 years, respectively 188. Additionally, they may also show more regulatory transcriptome expression patterns, and these transcription RNAs can affect gene transcription and translation 190, 191. In addition, telomere length and activity also show longer length and higher activity in healthy centenarians 192. Here, we take a simple example. In long-lived individuals, correlation analysis based on transcriptomics translation into proteomics has been reported. By developing a genome-wide precision metabolic modeling method with serum metabolomics and proteomics derived from transcriptomics, Li *et al.* concluded that elevated long- chain fatty acid beta-oxidation (FAO) is the most significant metabolic feature in centenarians 193. And they hypothesized that the elevated FAO would induce the consumption of long-chain fatty acids, and serum long-chain fatty acids appear at low levels in centenarians. Further serum metabolomics studies showed that 83 down-regulated metabolites (include 67 fatty acid-like metabolites) in centenarians. Thus, the unique metabolic characteristic of centenarians is the elevated FAO.

Multi-omics approaches also include the integration of metabolomics or proteomics with other omics, and the most typical composition is to integrate metabolomics with microbial diversity. Wilmanski *et al.* adopted three independent cohorts comprising over 9,000 individuals and discovered that the gut microbiome uniqueness measures in healthy individuals (>80 yrs) increased with age, and the plasma metabolome was characterized by raising level of tryptophan metabolites (3-indoxyl sulfate, 6-hydroxyindole sulfate, indoleacetate and indolepropionate), and these tryptophan metabolites were thought to have a positive impact on healthy aging 194. Moreover, the metabolites derived from tryptophan metabolism are essential for the gut bacteria to adapt to aging in human hosts. Studies have shown that tryptophan metabolites have been shown to be beneficial to health and extend survival in a number of animal models 195. In addition to the previously mentioned secondary bile acids 33, SCFAs 35 and tryptophan metabolites 194; exopolysaccharides 196 and polyamines 197 among other microbial-related metabolites, play crucial roles in health and longevity. Overall, beyond a certain age, long-lived individuals exhibit increasing gut microbiome uniqueness, which may result in a broader range of microbial-related metabolites entering the bloodstream to promote healthy aging.

In the past three years, a number of studies have examined the interaction between gut microbiome and metabolisms of long-lived individuals, such as the combination of fecal microbiota diversity and fecal metabolomics. Sato *et al.* found that centenarians have a distinct gut microbiome using fecal microbiome and bile acid metabolomics, and these gut bacteria were capable of generating unique secondary bile acids, such as isoallo-lithocholic acid (LCA) 33. This study reported for the first time that *Odoribacteraceae* relied on 5AR and 3βHSDH to produce isoalloLCA, and isoalloLCA could be used to against infections. Altogether, this study illustrates that gut bacteria from centenarians participated in a unique bile acid metabolism.

The effect of gut microbiome on host metabolism was analyzed by integrating fecal microbial diversity and blood metabolomics. Xu *et al.* compared the gut microbiome and blood metabolome of long-lived individuals (94-105 yrs) to that of offspring (50-79 yrs) in 116 Han Chinese families and found extensive metagenomic and metabolomic remodeling 35. The 16sRNA sequencing and metagenomic displayed nonagenarians and centenarians have greater microbial diversity than their descendants and have identified "longevous gut microbiota signature", including *Bifidobacterium* and *Blautia*. The targeted metabolomics showed that acetic acid, butyric acid, and propionic acid derived from *Bacteroides* over-expressed in centenarians. In general, nonagenarians and centenarians exhibited gut-related metabolic alterations. Our current research for centenarians analyzes serum metabolites and gut bacteria in fecal samples by the multi-omics integrating metabolomics, 16S rRNA, and metagenomics (unpublished data). Preliminary findings suggest that tryptophan metabolites such as 5-methoxyindole-3-acetic acid and indole-3-pyruvic acid were significantly elevated in healthy centenarians compared to frail centenarians. Furthermore, a significant positive correlation between elevated metabolite like oxindole and gut bacteria like* Christensenella* in centenarians have been observed. To sum up, several other omics studies in long-lived individuals are also worthwhile exploring, including those in the fields of genomics 198, 199 and microbiome 200.

## 5. Conclusion

In this review, we integrate longevity-related biomarkers discovered by metabolomics and proteomics and further categorize them based on different classes. The metabolites and proteins mentioned above are considered crucial signaling molecules for prolonging lifespan and alleviating age-related diseases. The mechanisms of longevity-related metabolites have been elucidated, especially for specific fatty acids like EPA, DHA, and SCFAs, which effect lifespan by reducing inflammation and activating the Nrf2 pathway. The mechanisms underlying the health benefits of the changes in certain metabolites are still largely unknown. For example, the mechanism of some isomers of secondary bile acids affects the body's immunity remains to be further studied. Additionally, the metabolic pathways and products of metabolites should also be considered. Some intermediates (such as kynurenic acid) have neuroprotective effects, which were produced from tryptophan. Regarding star proteins, APOE, FOXO, and SIRT are essential signaling proteins for cell survival, which can regulate cell proliferation, metabolism, inflammation, and stress responses by influencing multiple signaling pathways, including PI3K/Akt, NF-κB, etc. Moreover, post-translational modifications such as nitrosylation and glycosylation have important effects on the function and communication of proteins. The interaction between various modifications and star proteins creates a complex network that modulates cell survival to extend lifespan. Therefore, integrating candidate longevity-related biomarkers to conduct a "biomarker library of health and longevity" can further grasp the profile of centenarians or extreme longevity in humans and provide a theoretical foundation for anti-aging.

Metabolomics, proteomics, and multi-omics provide researchers with scientific methods to integrate information at different molecular layers, and further understand the origin and fates of small molecule substances. However, there are certain challenges and limitations in longevity cohort studies using metabolomics and proteomics, and the following interfering factors need to be carefully considered in study design and data interpretation.

Longevity cohort studies typically require large-scale population samples to obtain reliable results. Currently, several countries are conducting various longevity cohorts, such as the European Union Longevity Genetics Consortium, the New England Centenarian Study (NECS), and the Chinese Longitudinal Healthy Longevity Survey (CLHLS). Establishing longevity cohorts should consider the diversity of the population, including variations in age span, gender ratio, geographical location, and lifestyle, to ensure the accuracy and broad applicability of the results. For instance, in Chinese centenarians, females are disproportionately represented, necessitating the maintenance of gender balance to eliminate potential differences caused by gender in longevity studies. Due to the hereditary nature of certain longevity traits, such as the APOE e2 gene, understanding the participants' kinship is crucial for identifying genetic factors.

Appropriate analytical methods are crucial for different research objects based on the research question and sample characteristics. In metabolomics, untargeted and targeted metabolomics both have different advantages and disadvantages. It is worth noting that a certain degree of lipid metabolism dysfunction and neural functional damage happens during the aging process. Therefore, targeted metabolomics focusing on specific metabolites such as short-chain fatty acids, bile acids, and neurotransmitters can better reflect the physiological status of the elderly. In proteomics, general proteomics can provide a more comprehensive protein map for the longevity population. With an increase in age, protein homeostasis gradually declines and results in wrong translation modification such as nitrosylation and glycosylation. Consequently, post-translational modifications of proteins are considered crucial indicators affecting the function of proteins. The study of longevity cohorts based on untargeted metabolomics and general proteomics has been extensively reported. We regard that targeted metabolomics and PTM proteomics focusing on specific biomolecules will attract more attention in aging studies to discover more valuable longevity-related biomarkers, whether metabolites or proteins. Moreover, blood and fecal samples are commonly preferred for biomarker discovery due to their relatively easy access. If tissue-specific characteristics are exhibited in the liver or muscle tissues of centenarians, it potentially leads to obvious alterations in circulating blood metabolites. However, there are significant challenges in obtaining tissue samples such as liver and muscle from living individuals. We wish that better technology may have appeared in the future to offer the possibility to analyze the tissue or organ specificity of centenarians.

In terms of data analysis, standardization and uniformity are crucial steps to ensure experiment reproducibility and result comparability. Due to objective or unavoidable factors, such as gender differences, statistical or machine learning methods can be employed to establish an effective and reasonable model to eliminate the impact of non-research factors. Subsequently, validation through animal experiments is a crucial prerequisite for translating potential longevity biomarkers into clinical diagnosis and treatment. Some extreme longevity-related biomarkers should be replicated and verified in large cohorts or model animals such as nematodes, zebrafish, mice, and naked mole rats with an extreme lifespan.

Finally, combining and integrating different omics techniques has played an increasingly important role in scientific research. By effectively integrating multiple layers of biological information, we will be able to deepen our understanding of the biological mechanisms that regulate aging and prolong life. For example, metabolomics and proteomics are used in combination with other omics such as genomics, transcriptomics, and microbiomics to analyze the characteristics of biomarkers (metabolites, proteins, genes, transcription factors, microorganisms), and the connections of molecules mentioned above in functional annotations and signaling pathways in different clinical samples from long-lived populations. We look forward to integrating different omics and conducting association analyses in the future to discover biomarkers related to extreme longevity.

## Figures and Tables

**Table 1:** Summary of lipid species in longevity associated study.

Lipids	Populations	Group	Sample	Analyticaltechnique	Main findings	Year		Reference
Phospholipids	Centenarians	143 centenarians; aged; young	Serum	LC-MS/MS	PC, PC-O ↑ in centenarians and adults, compared to aged	2013		21
					PC, LPC, PC-O ↓ in centenarians, unchanging until 70 aged			
		98 centenarians; 196 aged	Serum	^1^H-NMR	PC, polyunsaturated PC-O, LPC, PE, PI ↑ in centenarians, compared to aged	2014		48
					saturated PC-O ↓ in centenarians, compared to elderly			
		27 centenarians; 31 aged, and 31 adults	Plasma	LC-MS/MS	PC, LPC, PE, LPE, PG, LPG, PA, PS ↑ in centenarians and adults compared to aged	2017		47
					LPE, LPI, PA, PG, PS ↓ in centenarians and adults, compared to aged			
		25centenarians; 22 aged, and 21adult.	Plasma	LC-MS/MS	total ether lipids, alkyl-PE, alkenyl-lipids/PE ↓ with age	2019		46
					alkyl-PC/LPC, alkenyl-PC/LPC ↑ in centenarians, compared to adults and aged			
	Elderly	143 centenarians; aged; young	Serum	LC-MS/MS	PC ↑ with age, LPC ↓ with age	2013		21
		offspring of nonagenarians; controls	Plasma	UPLC-MS	PC-O ↑ in offspring of nonagenarians, compared to controls	2013		29
					polyunsaturated PE ↓ in offspring of nonagenarians, compared to controls			
		150 subjects :30-49, 50-59, 60-79, and 90-99 years old	Plasma	LC-MS/MS	PC, PS ↓ with age	2016		47
		374 males (61.2±6.9); 838 females (60.7±6.6)	Plasma	LC-MS/MS	5 PC, 1 LPC, 3 PE ↑ with age; 3 PC, 1 LPC, 1 PE ↓ with age	2019		55
Sphingolipids	Centenarians	143 centenarians; aged; young	Serum	LC-MS/MS	SM, SM-OH ↓ in centenarians, compared to adults and aged	2013		21
		98 centenarians; 196 aged	Serum	^1^H-NMR	SM, Cer ↑ in centenarians, compared to aged	2014		48
		27 centenarians; 31 aged, and 31 adults	Plasma	LC-MS/MS	Cer ↑ in centenarians and adults, compared to aged	2017		47
					Cer, GM3, GlcCer ↓ in centenarians and adults, compared to aged			
Sphingolipids	Centenarians	25 centenarians; 22 aged, and 21adults	Plasma	LC-ESI-QQQ MS/MS	HexCer, Hex3Cer, GM3↑in centenarians, compared to aged and adults		2022	49
					Sulfatides, dhCer, SM (d18:0/22:0) ↓in centenarians, compared to aged and adults			
		15 centenarians; 15 aged, and 15 adults	Serum	Glyco/Sphingolipid LC-MS/MS	HexCer, Cer, GM3, SM ↑ in centenarians, compared to aged and adults		2022	50
					SM, S1P↓in centenarians, compared to aged			
					dhSM, dhCer, SM ↓ in centenarians, compared to aged and adults			
	Elderly	offspring of nonagenarians; controls	Plasma	UPLC-MS	SM↑in offspring of nonagenarians, compared to controls		2013	29
		374 males (61.2±6.9); 838 females (60.7±6.6)	Plasma	LC-MS/MS	24 SM ↑ with age, 1 SM ↓ with age		2019	55
Sterols	Centenarians	27 centenarians; 31 aged, 31 adults	Plasma	LC-MS/MS	CE ↑ in centenarians and adults, compared to aged		2017	47
	Elderly	150 subjects: 30-49, 50-59, 60-79, and 90-99 years old	Plasma	LC-MS/MS	15-keto-prostaglandin F2α ↓ with age		2017	47
		374 male (61.2±6.9); 838 female (60.7±6.6)	Plasma	LC-MS/MS	2 Steroids ↑ with age, 29 Steroids ↓ with age		2019	55
								

↓decreased levels,↑increased levelsPC Phosphatidylcholine, PE phosphatidylethanolamine, SM sphingomyelin, LPC lysophosphatidylcholine, LPE lysophosphatidylethanolamine, LPG lysophosphatidylglycerol, PA phosphatidic acid, PG phosphatidylglycerol, PS phosphatidylserine, LPI lysophosphatidylinositol, PC-O ether phosphatidylcholine, CE cholesteryl ester. Specific lipids have been named and classified according to Lipid Maps (http://www.lipidmaps.org)

**Table 2:** Some prominent proteins related with longevity study.

	Group	Sample	Analyticaltechnique	Description	Proteins	Year	Reference
Centenarians	centenarians (n=77, 105.7±3.6), centenarians' offspring (n=82, 71.2±9.3), controls (n=65, 70.6±7.8)	Serum	SOMAscan© technology	Proteins related to the APOE e2 allele	BIRC2, CEP57, VPS29, PSME1, TBCA,	2019	122
					UBA2, KMT2C, KIN, CKAP2		
				Proteins related to the APOE e4 allele	S100A13, LRRN1, APOE, C5orf38, CTF1, APOB, CRYZL1		
	Healthy centenarians (n=9, 100-103 yrs), controls (n=9, 67-81 yrs)	Plasma	TMT LC-MS/MS	↑in centenarians, compared to controls	CLEC3B, CRISP3, IGFALS, TAS1R3, TGFBI	2020	157
				↓in centenarians, compared to controls	AOPEP, C1S, CD14, CDKL1, CRTAC1		
	centenarians (n=77, 105.7±3.6), centenarians' offspring (n=82, 71.2±9.3), controls (n=65, 70.6±7.8)	Serum	SOMAscan© technology	↑in centenarians, compared to offspring and young	SFRP1, PTN, CHRDL1, QAGLN, GDF15, IGFBP2, COL28A1, SVEP, B2M FSTL3, NBL1, RSPO4, RNASE1, WFDC2, TNFRSF1B, IGFBP2, SMOC1, WISP2, IGFBP6, SPON1, DKK2, AKT2, HSP90AB1, STAT1, STAT3, SOST, DKK4, IGFBP7	2021	147
				↓in centenarians, compared to offspring and young	IGFALS, SERPINF2, ATP1B1, GDF11, CRP, CST3, GHR, IGFR, GHRL, IDE, SMAD3, FLT3, NUDT9, KLKB1, CKM		
	Semi supercentenarians (n=10, 106-109 yrs), centenarians (n=10, 100 yrs), healthy volunteers (n=10, 20-39 yrs)	Plasma	MALDI-TOF/MS	↑in Semi centenarians, compared to young	HP-β, A2 M, CLU	2010	95
	centenarians (105-114 yrs), aged (76-83 yrs), young (32-44 yrs)	Serum	Nitro-DIGE	S-nitrosation levels ↑in centenarians, compared to other groups	GSNOR	2020	163
				S-nitrosation levels ↓in centenarians, compared to other groups	SERPINA1, SERPINA3, CP, CERCAM, HP, TTR,		
					VDBP, IDLC1, TF, TRXR1		
Centenarians	Semi supercentenarians (mean 106.7 yrs), aged controls (mean 71.6 yrs), young controls (mean 30.2 yrs)	Plasma	LC-MS/MS; DSA-FACE; MALDI-TOF-MS	Glycosylation levels ↑ in Semi centenarians, compared to other groups	N-glycans (dHex1 Hex6 HexNAc5 NeuNAc3) ↑ in Semi centenarians	2015	164
					Multi-branched and highly sialylated N-glycans, as well as agalacto- and/or bisecting N-glycans		
				Glycosylation levels ↓ in Semi centenarians, compared to other groups	Biantennary N-glycans		
	Semi supercentenarians (106-109 yrs), aged controls (70-88 yrs), young controls (20-38 yrs)	Plasma	LC-MS/MS	Glycosylation levels ↑ in Semi centenarians, compared to other groups	Tri-antennary and sialylated N-glycans of haptoglobin at Asn207 and Asn211 sites were characterized in Semi centenarians	2018	175
Elderly	TwinsUK cohort (n=202, 65.30±6.92), replication Cohort (n=677, 76.96±7.06)	Plasma	SOMAscan© technology	↑with age	CHRDL1, CCDC80, PTN, FSTL3, TIMP1, MMP12,	2015	149
					CST3, IGFBP6, ROR1, THBS4, HA VCR2		
				↓with age	NA		
	men and women (n=240, 22-93 yrs)	Plasma	SOMAscan© technology	↑with age	GDF15, PTN, ADAMTS5, CGA FSHB, SOST,	2018	150
					CHRDL1, NPPB, EFEMP1, MMP12		
				↓with age	CTSV		
	offspring of parents with exceptional longevity (OPEL) (n=506, 74.5±6.1), offspring of parents with usual survival (OPUS) (n=519, 77.1±7.1)	Plasma	SOMAscan© technology	↑in OPEL, compared to OPUS	PTN, WISP2, CRDL1, TAGLN, RSPO1, GDF15, FBLN1, SMOC1, HE4, CST3, FSTL3, RNase1, sTREM-1, URB, NPPB, SREC-II	2020	151
				↓in OPEL, compared to OPUS	ERBB1/EGFR, SERPINF2		
	Long-lived men (≥90% expected survival for 12 years) (n=554, 78.5±3.1), Not long-lived men who died earlier (n=642, 76.4±2.9)	Serum	LC-IMS-MS	↓in long-lived men compared to non-long-lived men	C9, C7, CFD, CD5L, S100A9, MCAM, LGALS3BP, CSF1R, ALCAM, CRP, FCGBP, IGHG3, IGHM, FCGR3A, CD163, NRP1, GPLD1, B2 M, A2 M, MMP2, CST3, PTGDS, VWF, HPR, F2	2020	162

**Abbreviations:** A2 M, α2-macroglobulin; ADAMTS5, A disintegrin and metalloproteinase with thrombospondin motifs 5; ADH5/GSNOR, alcohol dehydrogenase 5/S-nitrosoglutathion; AKT2, RAC-beta serine/threonine-protein kinase; ALCAM, CD166 antigen; AOPEP, Aminopeptidase O; APOB, Apolipoprotein B; APOE, Apolipoprotein E; ATP1B1, Sodium/potassium-transporting ATPase subunit beta-1; B2 M, β2 microglobulin; BIRC2, Baculoviral IAP repeat containing 2; C1S, Complement C1s subcomponent; C5orf38, Chromosome 5 open reading frame 38; C7, Complement component C7; C9, Complement component C9; CCDC80, Coiled-coil domain- containing protein 80; CD14, Monocyte differentiation antigen CD14; CD163, Scavenger receptor cysteine-rich type 1 protein M130; CD5L, CD5 antigen-like; CDKL1, Cyclin-dependent kinase-like 1; CEP57, Centrosomal protein 57; CERCAM, Inactive glycosyltransferase 25 family member 3; CFD, Complement factor D; FSHB, Follitropin subunit beta; CHRDL1, Chordin-like protein 1; CKAP2, Cytoskeleton associated protein 2; CKM, Creatine kinase M-type; CLEC3B, Tetranectin; CLU, Clusterin precursor; COL28A1, Collagen alpha 1(XXVIII) chain; COL6A3, Collagen alpha 3(VI) chain; CP, Ceruloplasmin; CRISP3, Cysteine-rich secretory protein 3; CRP, C-reactive protein; CRTAC1, Cartilage acidic protein 1; CRYZL1, Crystallin zeta like 1; CSF1R, Macrophage colony-stimulating factor 1 receptor; CST3, Cystatin-C; CTF1, Cardiotrophin 1; CTSV, Cathepsin V; DKK2, Dickkopf-related protein 2; DKK4, Dickkopf-related protein 4; EFEMP1, Fibulin 3; ERBB1/EGFR, Epidermal growth factor receptor; F2, Prothrombin; FBLN1, EGF-containing fibulin-like extracellular matrix protein 1; FCGBP, IgG Fc-binding protein; FCGR3A, Low affinity immunoglobulin gamma Fc region receptor III A; FSTL3, Follistatin-related protein 3; GDF11, Growth/differentiation factor 11; GDF15, Growth/differentiation factor 15; GHR, Growth hormone receptor; GHRL, Appetite-regulating hormone; GPLD1, Phosphatidylinositol-glycan-specific phospholipase D; HA VCR2, Hepatitis A virus cellular receptor 2; HE4, WAP four-disulfide core domain protein 2; HP, Haptoglobin; HPR, Haptoglobin-related protein; HP-β, Haptoglobin β chain; HSP90AB1, Heat shock protein HSP 90-beta; IDE, Insulin-degrading enzyme; IGFALS, Insulin-like growth factor-binding protein complex acid labile subunit; IGFBP2, Insulin-like growth factor-binding protein 2; IGFBP6, Insulin-like growth factor-binding protein 6; IGFBP7, Insulin-like growth factor-binding protein 7; IGFR, IGF-like family receptor; IGHG3, Immunoglobulin heavy constant gamma 3; IGHM, Immunoglobulin heavy constant mu; IGLC1, immunoglobulin light chain 1; KIN, Kin17 DNA and RNA binding protein; KLKB1, Plasma kallikrein; KMT2C, Lysine methyltransferase 2C; LGALS3BP, Galectin-3-binding protein; LRRN1, Leucine rich repeat neuronal 1; MCAM, Cell surface glycoprotein; MMP12, matrix metallopeptidase 12; MMP2, matrix metallopeptidase 2; MSR1, Macrophage scavenger receptor types I and II;NBL1, Neuroblastoma suppressor of tumorigenicity 1; NPPB, N-terminal pro-BNP; NRP1, Neuropilin-1; NUDT9, ADP-ribose pyrophosphatase, mitochondrial; PCSK1, Neuroendocrine convertase 1; PGD2 synthase, Prostaglandin-H2 D-isomerase; PON1, Paraoxonase/arylesterase 1; PSME1, Proteasome activator subunit 1; PTGDS, Prostaglandin-H2 D-isomerase; PTN, Pleiotrophin; RNase 1, Ribonuclease pancreatic; ROR1, Tyrosine-protein kinase transmembrane receptor ROR1; RSPO1, R-spondin-1; RSPO4, R-spondin-4; S100A13, S100 calcium binding protein A13; S100A9, Protein S100-A9; SERPINA1, α1-antitripsin; SERPINA3, α1-antichimotripsin; SERPINF2, α2-antiplasmin; SFRP1, Secreted frizzled-related protein 1; SMAD3, Mothers against decapentaplegic homolog 3; SMOC1, SPARC-related modular calcium-binding protein 1; SOST, Sclerostin; SPON1, Spondin-1; SREC-II, Scavenger receptor class F member 2; STAT1, Signal transducer and activator of transcription 1-alpha/beta; STAT3, Signal transducer and activator of transcription 3; sTREM-1, Triggering receptor expressed on myeloid cells 1; SVEP, Sushi, von Willebrand factor type A, EGF and pentraxin domain-containing protein 1; TAGLN, Transgelin; TAS1R3, Taste receptor type 1 member 3; TBCA, Tubulin folding cofactor A; TF, serotransferrin; TGFBI, Transforming growth factor-beta-induced protein ig-h3; THBS4, Thrombospondin-4; TIMP1, Metalloproteinase inhibitor 1; TNFRSF1B, Tumor necrosis factor receptor superfamily member 1B; TRXR1, thioredoxin reductase 1; TTR, transthyretin; UBA2, Ubiquitin like modifier activating enzyme 2; URB, Unhealthy ribosome biogenesis protein homolog; VDBP, vitamin D-binding protein; VPS29, Vacuolar protein sorting-associated protein 29; VWF, von Willebrand factor; WFDC2, WAP four-disulfide core domain protein 2; WISP2, WNT1-inducible signaling pathway protein 2.
